# The Year-Book of Treatment for 1886

**Published:** 1887-05

**Authors:** 


### Reviews.

**The Year Book of Treatment for 1886.** A critical review for practitioners of medicine and surgery. Philadelphia: Lea Brothers. 1887.

This book, of about 300 pages, aims to give one a complete account of all the more important advances made in the treatment of disease, and also to furnish a review of the same by competent authorities. The contributors are all English, but they are most of them men eminent in the profession. They have sifted the medical literature of all countries for all matters relating to treatment that have been published during the year ending September 30, 1886. The work seems to us highly valuable, and it is full of suggestions and information to the practitioner.

